# Report of a Case of Cerebral Embolism

**Published:** 1890-06

**Authors:** C. D. Roy

**Affiliations:** Charity Hospital


### Clinical Department.

### *REPORT OF A CASE OF CEREBRAL EMBOLISM.

BY C. D. ROY, M. D., CHARITY HOSPITAL.

The case presented adds nothing new to the pages of clinical medicine, but is one which proved to be so unique in its character, and so rapidly fatal in its termination as to be worthy of recording, in order to fix it upon the minds of practitioners as a condition not infrequently met with:

J. M., male, age 26, a stout, robust looking man, was admitted into the hospital and immediately transferred to the ward with which I was connected. On coming in, the only objective or subjective condition for which he wished to be treated was paresis or partial paralysis of the right arm. Said he had been a very hard drinker, and one day, a month previous, he had been seized with a convulsion on the street, became unconscious, and was carried home in that state. On recovering consciousness he found his right arm paralyzed, which improved gradually. Never had a convulsion before, nor was it hereditary in his family. Had always been remarkably healthy, having only had a slight attack of rheumatism. His pulse was fair, though somewhat diminished in force and reseliency. On examining his heart with the stethescope, there was found two murmurs, viz.: mitral regurgetant and aortic stenotic. He was placed on the salicylates for his rheumatic tendency, tonics were administered, and the tincture of strophanthus for the regulation of, and the increase in the force of the heart's action, the stage of dilatation not having been reached. The treatment acted admirably, and on the seventh morning he desired to leave the hospital. Unexpectedly while taking a bath the same morning, he was suddenly seized with total hemiplegia of the right side. There was also loss of sensation with profound coma, from which he was never aroused. The cause could hardly be mistaken, and the diagnosis of cerebral embolism was made, although no post-mortem was allowed upon the body. The embolus must have been one of large size in the middle meningeal artery, for the symptoms showed the complete involvement of the internal capsule. Congestion and œdema of the lungs soon followed. Death terminated at four o'clock, all efforts to carry him through the initial shock being unavailing. Some temporary benefit was received by the injection subcutaneously of two minims of a one per cent solution of nitroglycerine every hour, acting as it did by lessening the pulmonary congestion. It has been my observation and belief that many of the monoplegias are due to an embolism of some of the vessels in the motor area. While there was evidently an embolus carried from the heart, the blood vessels themselves were in a favorable condition for the formation of a thrombus. The radical artery felt hard, and failed to give that rebound of the normal pulse, evidently showing some changes in the blood vessels themselves, caused no doubt by the habitual use of alcohol.

